# Study on Mutual Information and Fractal Dimension-Based Unsupervised Feature Parameters Selection: Application in UAVs

**DOI:** 10.3390/e20090674

**Published:** 2018-09-05

**Authors:** Xiaohong Wang, Yidi He, Lizhi Wang

**Affiliations:** 1 School of Reliability and Systems Engineering, Beihang University, Beijing 100191, China; 2 Institute of Unmanned System, Beihang University, Beijing 100191, China; 3 Key Laboratory of Advanced Technology of Intelligent Unmanned Flight System of Ministry of Industry and Information Technology, Beihang University, Beijing 100191, China

**Keywords:** unsupervised feature selection, feature extraction, mutual information, fractal dimension, subspace learning algorithm

## Abstract

In this study, due to the redundant and irrelevant features contained in the multi-dimensional feature parameter set, the information fusion performance of the subspace learning algorithm was reduced. To solve the above problem, a mutual information (MI) and fractal dimension-based unsupervised feature parameters selection method was proposed. The key to this method was the importance ordering algorithm based on the comprehensive consideration of the relevance and redundancy of features, and then the method of fractal dimension-based feature parameter subset evaluation criterion was adopted to obtain the optimal feature parameter subset. To verify the validity of the proposed method, a brushless direct current (DC) motor performance degradation test was designed. Vibrational sample data during motor performance degradation was used as the data source, and motor health-fault diagnosis capacity and motor state prediction effect ware evaluation indexes to compare the information fusion performance of the subspace learning algorithm before and after the use of the proposed method. According to the comparison result, the proposed method is able to eliminate highly-redundant parameters that are less correlated to feature parameters, thereby enhancing the information fusion performance of the subspace learning algorithm.

## 1. Introduction

With the development of scientific and technological research, research objects in various fields such as mechanical engineering, data mining, image processing, information retrieval, and genome engineering are becoming increasingly complex. Therefore, the volume of experimentally acquired data, such as product fault data, genetic data, and high-definition image information, has also increased exponentially, as has the number of feature dimensions [1]. Multidimensional feature parameters usually exhibit sparsity. The information between any feature parameters overlaps and complements each other while there are various problems facing data description, such as poor overall identification, heavy calculation, difficulty in visualization, and incorrect conclusions. To this end, subspace learning algorithms, such as Principal Component Analysis (PCA) [2], Kernel Principal Component Analysis (KPCA) [3], Linear Discriminant Analysis (LDA) [4], Locality Preserving Projections (LPP) [5], and Locally Linear Embedding (LLE) [6], have gradually been applied to information fusion of multidimensional feature parameters. However, these methods have failed to consider the possible redundant and irrelevant feature parameters in the multidimensional feature parameter space, of which redundant features might reduce the information fusion efficiency of the subspace learning algorithms and irrelevant features might undermine the performance of subspace learning algorithms. This might eventually lead to reduced information fusion performance of the aforementioned subspace learning algorithms and affect their precision and stability [7].

With the feature selection method, the optimal feature parameter subset can be obtained and the redundant and irrelevant features in it can be eliminated with minimum information loss, thereby enhancing algorithm performance and saving running time [8]. In view of the problems above, a feature selection method was used to eliminate the redundant and irrelevant features in the feature parameter subset. Considering that the data in engineering practice and application is mostly unlabeled, feature selection should be made using the unsupervised method since it does not require data labels and select the feature subset that contain the key properties of the original feature set from a data perspective [1]. Currently, unsupervised feature selection method is made using a combined method of the search algorithm (such as genetic algorithm [9,10] and ant colony optimization [11,12]) and feature parameter subset evaluation criterion (fractal dimension [13,14] and rough set theory [15]). However, such a method might result in reduced precision of subsequent algorithms since they are troubled by heavy calculations, long running times and high time complexities ***O***(2*^n^*) [7]. In certain instances, there might not be an optimal solution.

In information theory, entropy is a measure of uncertainty in a physical system [16,17]. Based on this definition, the information shared by two things, namely the interdependence between them, can be characterized by mutual information (MI). Thus, MI is an effective tool of measuring feature relevance and redundancy. Similar with the “Minimum Redundancy and Maximum Relevance (mRMR)” [18] of the supervised method, the basic idea of a MI-based unsupervised method also takes the redundancy and relevance of any feature parameter into overall consideration. As the quantitative index of fractal theory, fractal dimension (FD) measures the similar structures between entirety and locality [13,14,19,20,21], for which the similar properties between the feature parameter set and its subsets can be evaluated using FD. In this study, a mutual information and fractal dimension-based unsupervised feature selection (UFS-MIFD) method was developed based on the characteristics of MI and FD. To begin with, the linear ordering of feature parameters by importance was conducted as per their maximum “relevance” to the feature parameter set and minimum “redundancy” ordered feature set. The optimal feature parameter subset was selected from the ordered feature parameter set by using FD as the criterion of feature subset evaluation. Compared with existing feature selection algorithms, this method not only featured linear time complexity, significantly shortened running time, and greatly reduced searches, but the redundant and irrelevant features in the feature parameter set were also eliminated.

**Figure 1:**
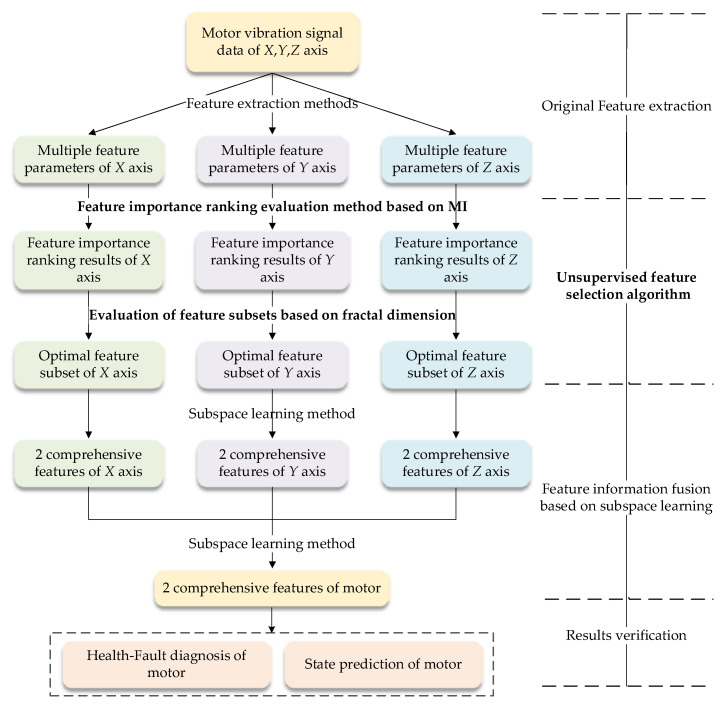
Paper flowchart.

Multi-rotor Unmanned Aerial Vehicles (UAVs) represent a new type of UAV with prominent features, such as simple mechanical structure, convenient use and maintenance, vertical take-off and landing, and rapid release, which make multi-rotor UAVs studied and applied in many fields such as military surveillance, power line inspection, pesticide spraying, and other fields such as express delivery in recent years. The brushless direct current (DC) motor is a power supply unit in multi-rotor UAVs, and its safety and reliability directly affects the reliability level of the multi-rotor UAV itself. Therefore, in this paper, a brushless DC motor performance degradation test was designed to acquire vibrational signals, which are used as the data source to verify the proposed method from the perspectives of fault diagnosis and state prediction. The UFS-MIFD method was investigated as shown in Figure 1. The rest if this paper is organized as follows: the process of UFS-MIFD is presented in Section 2. The brushless DC motor, the test method and process, and vibration signal acquisition and analysis are introduced in Section 3. In Section 4, the original feature parameter set is extracted based on motor vibration signals; the implementation of UFS-MIFD algorithm is also introduced. In Section 5, the validity of the proposed UFS-MIFD is verified based on the information fusion result of the output subspace learning algorithm obtained in Section 4 from the perspectives of the motor health-fault diagnosis effect and motor state prediction. Conclusions of this study and prospects for further studies are presented in Section 6.

## 2. Mutual Information and Fractal Dimension-Based Unsupervised Feature Parameters Selection Method

### 2.1. Theoretical Basis

#### 2.1.1. Mutual Information (MI)

Mutual information is defined based on information entropy. It measures the interdependence between two features, which means it represents the information shared by both features. Suppose that there is a feature parameter set *F* comprising *n* feature parameters $f_{1},f_{2},\cdots ,f_{n}$. According to information entropy theory, the mutual information between feature parameters $f_{i}$ and $f_{j}$ can be defined as:

(1) $$I\left(f_{i}|f_{j}\right)=H\left(f_{i}\right)\;-\;H\left(f_{i}|f_{j}\right)=I\left(f_{j}|f_{i}\right)\;$$

where $H\left(f_{i}\right)$ is the information entropy of feature $f_{i}$ (see Equation (2)) [16,17]; $P\left(f_{i}\right)$ is the probability of feature variable $f_{i}$ taking different probable values, which measures the uncertainty of the value of $f_{i}$; $H\left(f_{i}|f_{j}\right)$ is the conditional entropy (see Equation (3)), which means the uncertainty of $f_{i}$ when the value of another feature $f_{j}$ is known:

(2) $$H\left(f_{i}\right)=-\sum_{f_{i}}P\left(f_{i}\right)\log P\left(f_{i}\right)\;$$

(3) $$H\left(f_{i}|f_{j}\right)=-\sum_{f_{j}}P\left(f_{j}\right)\sum_{f_{i}}P\left(f_{i}|f_{j}\right)\log P\left(f_{i}|f_{j}\right)\;$$

In fact, however, the relevance between the feature parameters in the feature parameter set and their redundant features cannot be measured directly by MI, for which the mRMR criterion in the supervised method is required to measure the relevance and redundancy of features.

#### 2.1.2. Fractal Dimension

Fractals are ubiquitous in Nature. Due to the limited data points in the data set, the dataset shows fractal features only within a certain scale range, namely when the local distribution and global distribution of the dataset share similar structure or properties. In this case, it can be analyzed using fractal theory [13,14,19,20,21]. FD is the quantitative index of fractal theory. There are a variety of methods that can be used to calculate the FD of the dataset, of which the box-counting method is easy to implement and widely used. Therefore, FD was also calculated using box-counting method in this paper. With this method, the dataset is covered using a hypercube with a scale of *ε*, thereby obtaining the FD of the dataset. In non-scaling interval $\left[\varepsilon_{1},\varepsilon_{2}\right]$, the FD of feature parameter set *X* with *N* dimensions can be calculated using the following Equation (4):

(4) $$D(X)=\underset{\varepsilon \to 0}{\lim}\frac{\ln N(\varepsilon )}{\ln\left(1/\varepsilon \right)}\;$$

where *ε* is the side length of the hypercube; *N*(*ε*) is the minimum number of hypercubes with a side length of *ε* that cover *X*. The points are plotted in the double logarithm coordinates based on the equation above. The least squares method is used to fit non-scaling interval $\left[\varepsilon_{1},\varepsilon_{2}\right]$, thus obtaining the FD of the dataset.

### 2.2. UFS-MIFD Method

The fundamental theories mentioned in Section 2.1 were extended in this paper. A UFS-MIFD algorithm was developed by drawing from mRMR of the supervised method. To begin with, the relevancy, conditional relevancy and redundancy between feature parameters [7] were defined and calculated. With overall consideration, the mRMR criterion for feature parameter importance ordering was obtained, based on which the importance ordering of feature parameters contained in the feature parameter set was conducted. The less important a feature parameter was, the lower the relevancy between the parameter and the overall feature parameter set and the higher the redundancy was. Next, the feature subsets of the ordered parameter set were selected as per the FD-based feature subset evaluation criterion, thereby eliminating the feature parameters with lower relevancy and high redundancy from the feature parameter set. The algorithmic process is as follows:

First, the importance ordering of various feature parameters in the *n*-dimensional original feature parameter set $F=[f_{1},f_{2},\cdots ,f_{n}]$ was conducted stepwise. The ordered feature set was supposed as *G* and left empty.

***Step 1:*** The average MI between the whole feature parameter set *F* and every feature $f_{i}\left(i=1,2,\cdots ,n\right)$ was calculated using Equation (5):

(5) $$score\left(f_{i}\right)=\frac{1}{n}\sum_{j=1}^{n}I\left(f_{i};f_{j}\right)$$

Thus, the first important feature in *G* could be $g_{1}=f_{l_{1}}$, where $l_{1}=\arg\underset{1\;\leq \;i\;\leq \;n}{\max}\left\{score\left(f_{i}\right)\right\}$. This feature was able to minimize the uncertainty of the rest of features in *F*.

***Step 2:*** To obtain the second important feature in *G*,$F=[f_{1},f_{2},\cdots ,f_{n}]$ was replaced by $F=[f_{1},f_{2},\cdots ,f_{j},\cdots ,f_{n-1}]$. In this case, feature $f_{j}$, where j=1,2,⋯,n−1, was selected randomly from *F* to calculate its relevancy Rel(fj) with *F*, the conditional relevancy Rel(g1|fi) between $g_{1}$ in *G* and $f_{j}$, and the redundancy Red(fj;g1) of $f_{j}$ with respect to $g_{1}$, of which Rel(fj) was defined as the average MI between $f_{j}$ and *F* [7]:

(6) Rel(fj)=1n∑k=1n−1I(fj;fk)=1n(H(fj)+∑1 ≤ k ≤ n, j ≠ knI(fj;fk)) 

where $H\left(f_{j}\right)$ signifies the information $f_{j}$ contains; $\sum_{1\;\leq \;k\;\leq \;n,\;j\;\neq \;k}^{n}I\left(f_{j};f_{k}\right)$ means the information shared by $f_{j}$ and other parameters in *F*. The larger $\sum_{1\;\leq \;k\;\leq \;n,\;j\;\neq \;k}^{n}I\left(f_{j};f_{k}\right)$ was, the less the new information the other parameters could provide. Therefore, if the feature parameter with the largest Rel(fj) was selected, there would be the least information loss in the corresponding parameter set. The conditional relevancy Rel(g1|fi) between $f_{j}$ and $g_{1}$ could be defined as [7]:

(7) Rel(g1|fj)=H(g1|fj)H(g1)Rel(g1) 

The redundancy Red(fj;g1) of $f_{j}$ with respect to $g_{1}$ could be defined as follows [7]:

(8) Red(fj;g1)=Rel(g1)−Rel(g1|fj) 

Thus, the importance evaluation criterion *E* for feature parameter $f_{j}$ could be obtained by taking the relevance between $f_{j}$ and *F* and the redundancy of $f_{j}$ with respect to *G* into overall consideration:

(9) E(fj)=Rel(fj)−maxg1∈G{Red(fj;g1)} 

Suppose that $l_{2}=\arg\underset{1\;\leq \;j\;\leq \;n-1}{\max}\left\{E\left(f_{j}\right)|f_{j}\in F\right\}$, the second feature in *G* was $g_{2}=f_{l_{2}}$.

***Step 3:*** Similarly, the original *F* was replaced by $F=[f_{1},f_{2},\cdots ,f_{j},\cdots ,f_{n-p+1}]$ to obtain the *p*-th important feature in *G*. In this case, feature $f_{j}$, where j=1, 2, ⋯, n−p+1, was selected randomly from *F*. The relevance Rel(fj) between $f_{j}$ and *F*, the conditional relevance Rel(gm|fj) between $g_{m}$ in *G* and $f_{j}$, and the redundancy Red(fj;gm) of $f_{j}$ with respect to $g_{m}$, where, were calculated using Equations (6)–(8). Thus, the importance evaluation criterion *E* for feature parameter $f_{j}$ could be obtained by taking the relevance between $f_{j}$ and *F* and the redundancy of $f_{j}$ with respect to *G* into overall consideration:

(10) E(fj)=Rel(fj)−maxgm∈G{Red(fj;gm)} 

Suppose that $l_{r}=\arg\underset{1\;\leq \;j\;\leq \;n-p+1}{\max}\left\{E\left(f_{j}\right)|f_{j}\in F\right\}$, *p*-th feature in *G* was $g_{p}=f_{l_{r}}$.

***Step 3*** was repeated until all the feature parameters in the original feature parameter set *F* were ordered by their importance, that is, the ordered feature parameter set *G* was obtained.

***Step 4:*** On that basis, the subsets of the ordered feature parameter set *G* were selected using the FD-based feature parameter subset evaluation criterion proposed in this study. The main idea was that the feature parameter subsets wherein the difference between the partial fractal dimension and overall fractal dimension satisfied a certain threshold were reserved by eliminating the feature parameter that had the least influence on the feature parameter set once at a time. The steps are given as follows:

(1) The FD of *N*-dimensional ordered feature parameter set *G* was calculated, denoted as *frac*(*G*).
(2) With the *N*-th feature parameter $g_{N}$ eliminated from *G*, there were *N* − 1 feature parameters, which constituted a new feature parameter subset *S_N−_*_1_. To distinguish between *S_N−_*_1_ and *frac*(*G*), the fractal dimension *frac*(*S_N−_*_1_) of *S_N−_*_1_ was named the local fractal dimension. According to calculation, *r* = *frac*(*G*) − *frac*(*S_N−_*_1_). If |r|≤η (η was the threshold parameter), *S_N−_*_1_ was considered similar with *G*. Although the *N*-th feature parameter had been eliminated, it would not make a difference to *G*, which suggested that the *N*-th feature parameter was a highly redundant parameter that was less correlated to *G*.
(3) Let *frac*(*G*) = *frac*(*S_N−_*_1_), *G* = *G*−{$g_{N}$}, and *N* = *N* − 1. The calculation in step (2) was continued until |r|>η. At this point, the feature parameter subset was the optimal feature parameter subset.

**Figure 2:**
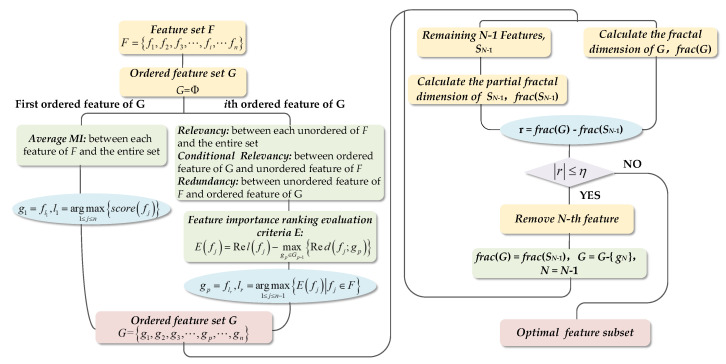
Process of the mutual information and fractal dimension-based unsupervised feature selection algorithm.

The flow diagram of the proposed method is shown in Figure 2.

## 3. Motor Vibration Data Acquisition and Signal Analysis

This section may be divided by subheadings. It should provide a concise and precise description of the experimental results, their interpretation as well as the experimental conclusions that can be drawn.

**Figure 3:**
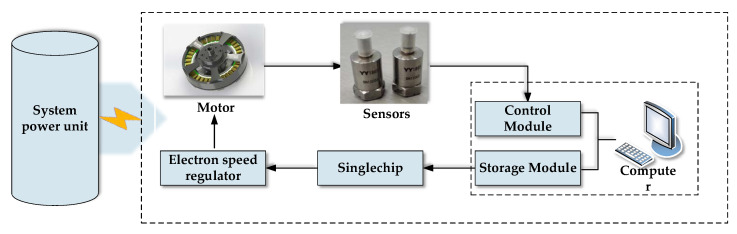
Motor degradation test system.

In this paper, the power motor (the U8 disc type brushless DC motor from T-MOTOR) of an unmanned multi-rotor gyroplane was taken as the research object, based on which a test was designed to monitor the vibrational signals during motor operation. Vibrational signals were used as the sample data for verifying the proposed method and motor performance degradation. The test system is shown in Figure 3. The working process was: the single chip microcomputer that was controlled by the control module of the computer sent pulse-width modulation (PWM) signals to the digital speed regulator that controlled motor operation. Motor vibration signals along *X, Y* and *Z*-axes were acquired using the acceleration sensor, which were then stored in the storage module of the computer. The modules of the test system were powered using the system power unit.

This motor performance degradation test was carried out at a 22.2 V rated operating voltage and 100% throttle. The test conditions are shown in Table 1.

**Figure 4:**
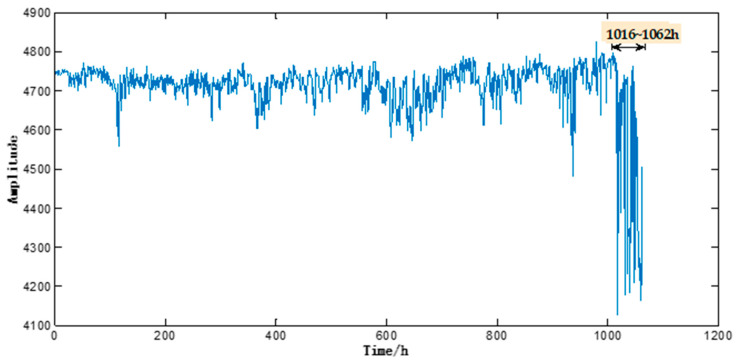
Operating states of the testing motor.

**Figure 5:**
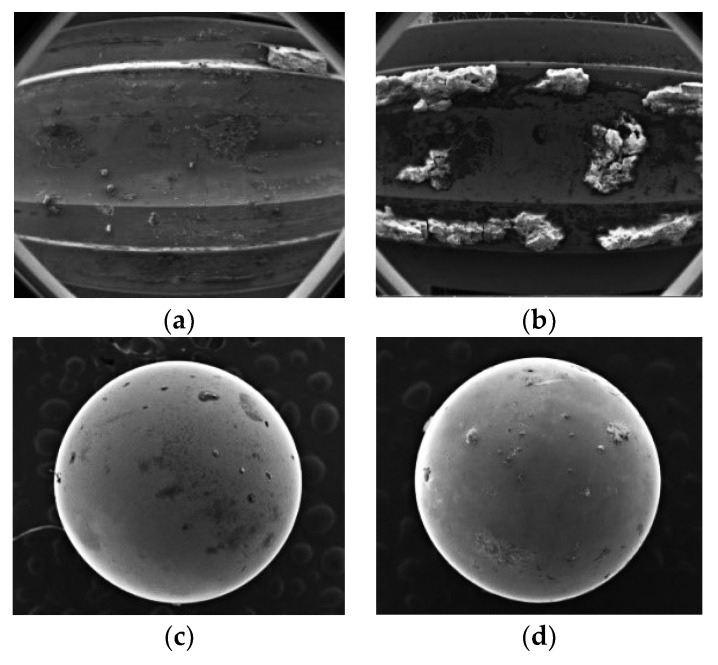
Scanning Electron Microscopy (SEM) images of the motor bearing. (**a**) Outer surface of inner bearing race, (**b**) Inner surface of outer bearing race, (**c**) Bearing ball 1, (**d**) Bearing ball 2.

This motor performance degradation test lasted 1062 h, during which the 1416 sample signals (each signal lasted 0.5 s) were captured and recorded at a time interval of 45 min from *X*, *Y* and *Z*-axes. As shown in Figure 4, the motor sample under test ran basically stably during 0–1016 h, but an abrupt change of its operating state was observed during 1017–1062 h. Such abnormality continued without any sign of weakening or disappearing. As shown in Figure 5, electron microscopy suggested noticeable abrasion on the surfaces of the inner and outer bearing races and bearing balls of the motor sample under test, which indicated that the motor sample under test had failed. Therefore, the motor vibration data acquired during 0–1016 h was taken as the initial input data.

## 4. Motor Vibration Feature Extraction and Selection

The features of vibrational data acquired during motor operation were extracted from the perspectives of degradation description and life evaluation. In this study, the feature parameter extraction methods included time domain feature parameter extraction method [22], frequency domain feature parameter extraction method [23], wavelet packet band energy (WPBE) feature parameter extraction method [24], and entropy measure-based feature parameter extraction method [25]. The commonly used time domain feature parameters were mean value, variance (VAR), peak, root mean square (RMS), skewness, kurtosis, pulse, margin, waveform, and peak value; the commonly-used frequency domain feature parameters included gravity frequency (GF), mean-square frequency (MSF), and frequency variance (FV). Entropy-based feature parameters included amplitude spectrum entropy (ASE) and Hilbert marginal spectrum entropy (HMSE).

**Figure 6:**
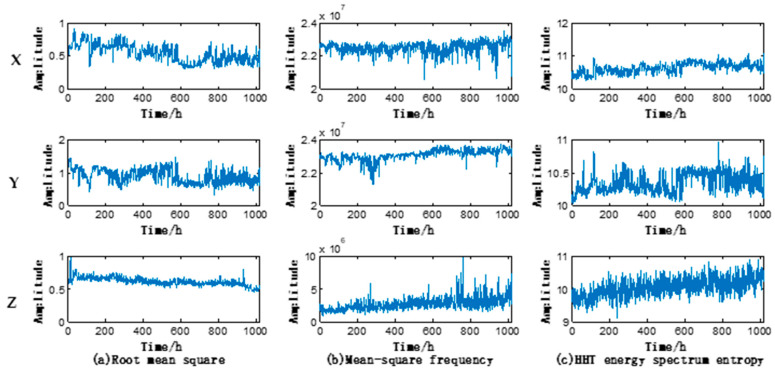
Root mean square (RMS), mean-square frequency (MSF), and Hereditary hemorrhagic telangiectasia (HHT) energy spectrum entropy along each axis.

With the aforementioned feature parameter extraction method, the feature parameters of vibration data along *X*, *Y*, and *Z*-axes were extracted, thus obtaining the triaxial 24-dimensional feature parameters. The triaxial operating state features of the motor under test are shown in Figure 6 (taking RMS, MSF, and Hereditary hemorrhagic telangiectasia (HHT) energy spectrum entropy as an example). It could be seen that the feature parameters along *X*, *Y*, and *Z* axes differ from each other.

**Figure 7:**
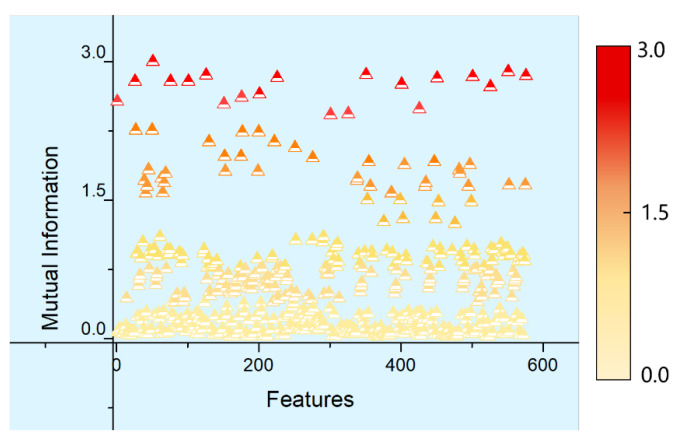
Mutual information of various feature parameters along *X*-axis.

According to the definition of mutual information given in Section 2.1, the information shared by the feature parameters along *X*, *Y*, and *Z*-axes was measured using the mutual information index. The distribution of mutual information between various feature parameters is shown in Figure 7 (taking *X*-axis as an example), where the horizontal axis means the arbitrary combination of two 24-dimensional feature parameters along the *X*-axis. Thus, there are 576 combinations. Each point represents the mutual information between any two feature parameters in the 24-dimensional feature parameter set of the motor along the *X*-axis, with its numerical values shown by gradient colors. According to calculations, the mutual information between various feature parameters along the *X*-axis was larger than 0 and the numerical value of mutual information between any two feature parameters differed from each other, which indicated that the information between various feature parameters along *X*-axis overlapped each other with certain relevance. Similarly, calculations also suggested that the mutual information, with different numerical values, between various feature parameters along *Y* and *Z*-axes was also larger than 0. This also evidenced that the information between various feature parameters along the *Y* and *Z*-axes overlapped each other, with certain relevance between them.

**Figure 8:**
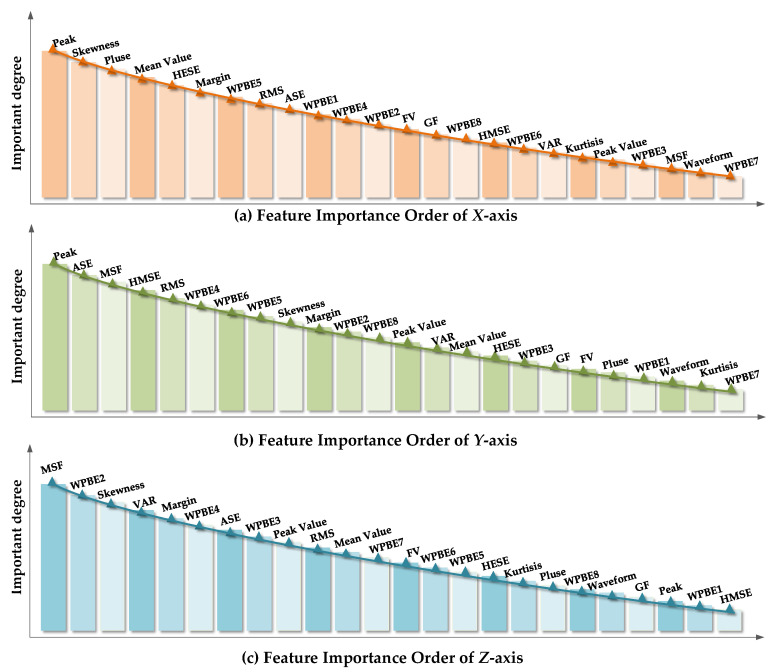
Importance of various feature parameters along *X*, *Y*, and *Z*-axes. (**a**) Feature importance order of *X*-axis; (**b**) Feature importance order of *Y*-axis; (**c**) Feature importance order of *Z*-axis.

The UFS-MIFD algorithm proposed in Section 2.2 was used to order the original feature parameter set of the motor under test along *X*, *Y*, and *Z*-axes by importance. The results of the importance ordering of feature parameters along the three axes, namely *G_X_*, *G_Y_*, and *G_Z_*, are shown in Figure 8a–c, respectively.

It can be seen that the peak was the most important feature parameter in the original feature parameter set along the *X* and *Y*-axes while MSF was the most important feature parameter in the original feature parameter set along the *Y*-axis. Figure 8 also suggests significant differences between various feature parameters in the feature parameter sets along the three axes which reflected the difference between feature parameters along various axes.

The important orders feature parameters of the motor under test along the *X*, *Y* and *Z*-axes, namely *G_X_*, *G_Y_*, and *G_Z_*, were evaluated based on the feature parameter subset evaluation criterion mentioned in the Step 4 of Section 2.2, where the threshold parameter η=0.05. Eventually, the feature subset *S*_X_ of the *X*-axis contained the first 17 feature parameters of *G_X_*. Similarly, the feature subset *S_Y_* contained the first 16 feature parameters of *G_Y_*; the feature subset *S_Z_* contained the first 13 feature parameters of *G_Z_*, as shown in Table 2.

**Figure 9:**
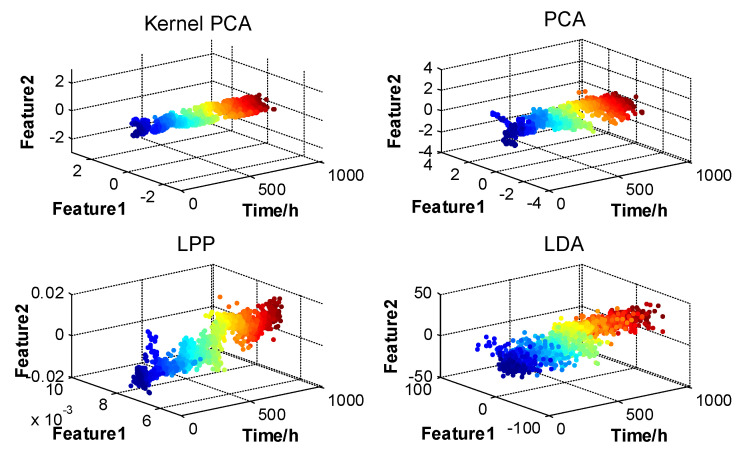
Final fusion results of the feature parameters of motor operating states.

It is generally believed that major feature information can be covered by the first two-dimensional feature parameters fused by the subspace learning method. In this study, the operation state information of the motor under test was fused by the process of feature information fusion based on subspace learning shown in the third part of Figure 9 using subspace learning methods, such as KPCA [3], PCA [2], LPP [5], and LDA [4]. Thus, the two-dimensional integrated feature parameters of the motor operating states were obtained. The final fusion result is shown in Figure 9. It could be seen that the motor operating degradation paths described by KPCA, PCA, and LPP fluctuated less than that by LDA, which evidenced that the KPCA, PCA, and LPP performed better in describing the motor operating state than LDA.

## 5. Results Verification and Analysis

### 5.1. Health-Fault Diagnosis of Motor

**Figure 10:**
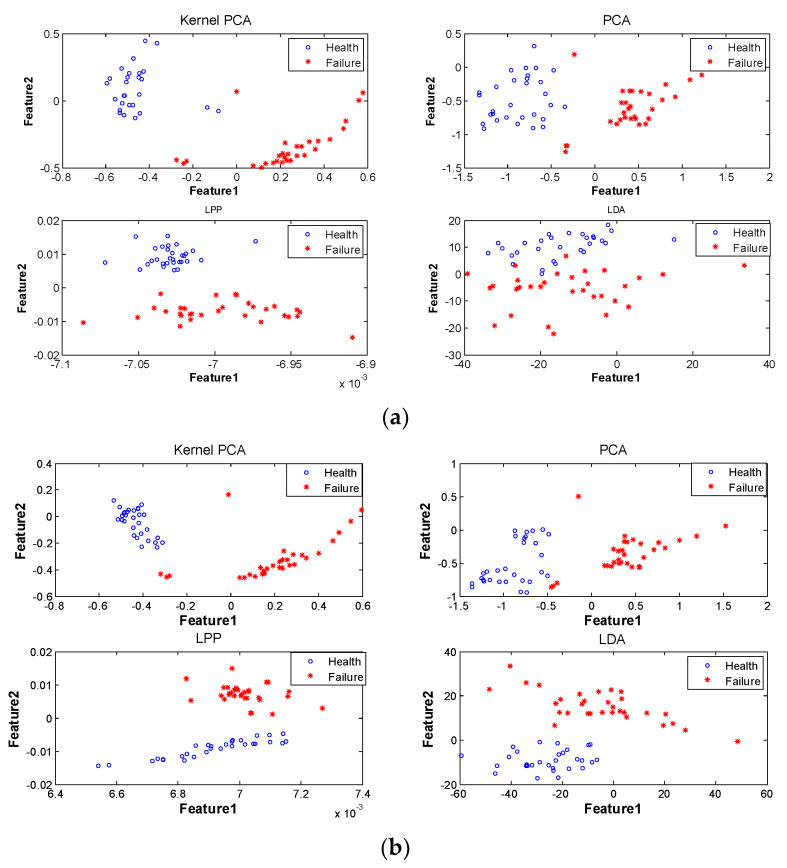
Comparison of the health-fault state diagnosis results based on the integrated feature parameters before and after the use of mutual information and fractal dimension-based unsupervised feature selection (UFS-MIFD). (**a**) Before the use of UFS-MIFD. (**b**) After the use of UFS-MIFD.

As shown in Figure 10, the “health-fault” states of the motor under test were identified based on the feature fusion result of motor operating state obtained in Section 4. Before the use of UFS-MIFD, information fusion of the original feature parameter set was made using the aforementioned four subspace learning methods. The result of health-fault states obtained based on the information fusion according to the two-dimensional integrated feature parameters *F*_1_ and *F*_2_ is shown in Figure 10a. Information fusion of the optimal feature parameter subsets *S_X_*, *S_Y_*, and *S_Z_* was made using the aforementioned four subspace learning methods after the use of UFS-MIFD. The result of “health-fault” states obtained based on the information fusion according to the two-dimensional integrated feature parameters *F*_1_* and *F*_2_* is shown in Figure 10b. It can be seen that an even better health-fault state diagnosis could be observed using two-dimensional integrated motor parameters. In the following sections, quantitative evaluation of the diagnostic result will be made.

Quantitative evaluation of the health-fault state diagnosis shown in Figure 10 was carried out using cluster evaluation index *D*. The form of evaluation index *D* is shown as follows [26]:

(11) $$D=\frac{tr(S_{w}{}_{{}_{1}})+tr(S_{w}{}_{{}_{2}})}{tr(S_{b})}\;$$

where $S_{w}{}_{{}_{1}}$ and $S_{w}{}_{{}_{2}}$ represent the within-class scatter matrices (covariance matrices) of health and fault state samples, which can be used to characterize the distribution of various state sample points around the mean value; $tr(S_{w}{}_{{}_{1}})$ and $tr(S_{w}{}_{{}_{2}})$ are the traces of the within-class scatter matrices of the two state samples, and a smaller value means more concentrated internal distribution of various state samples and better aggregation; $S_{b}$ is the between-class scatter matrix of health and fault state samples, which characterizes the distribution of various state samples in the space. The expression of $S_{b}$ is given as follows:

(12) $$S_{b}=\sum_{i=1}^{c}P(i)({\vec{M}}_{i}-{\vec{M}}_{0}){\left({\vec{M}}_{i}-{\vec{M}}_{0}\right)}^{T}\;$$

where P(i) is the prior probability of *i*-th class state samples; ${\vec{M}}_{i}$ is the mean vector of the *i*-th class state samples; ${\vec{M}}_{0}$ is the overall mean vector of state samples of class *c*, and ${\vec{M}}_{0}=\sum_{i=1}^{c}P(i){\vec{M}}_{i}$; $tr(S_{b})$ is the trace of the between-class scatter matrix of the two classes of state samples. A larger $tr(S_{b})$ suggests more scattered distribution of various state samples, which better helped to distinguish motor states. Therefore, the health-fault state diagnosis evaluation index *D* could be expressed as the ratio between the sum of the traces of within-class scatter matrices of the two classes of state samples and the sum of the traces of between-class scatter matrices of the two classes of state samples. A smaller *D* suggested better efficacy of the subspace learning algorithm in distinguishing the health-fault states. The evaluation result of the health-fault state diagnosis effect shown in Figure 10 is given in Table 3.

It could be seen from Table 3 that the information fusion performance of the four subspace learning methods—KPCA, PCA, LPP, and LDA—was found improved after using UFS-MIFD for feature selection, which enabled them to distinguish the motor health-fault states more correctly and clearly. In addition, the degree of performance enhancement is related to the selection of the subspace learning algorithm.

### 5.2. State Prediction of Motor

**Figure 11:**
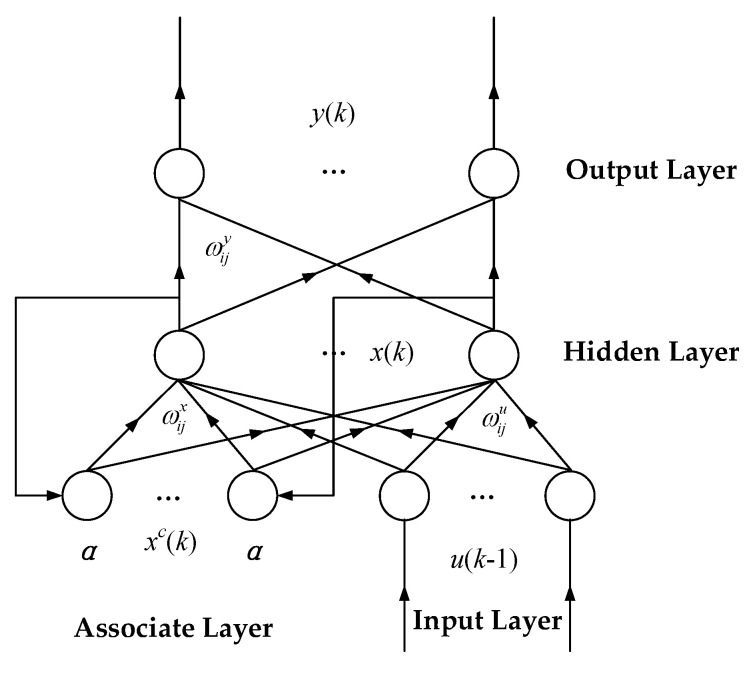
Elman neuron network structure.

Motor state prediction was conducted using the Elman neuron network prediction method based on the discussion above. As shown in Figure 11, Elman is a typical dynamic recurrent neuron network. Unlike common neuron network structures, Elman additionally contains an association layer that is designed to memorize the output value of the hidden layer at the previous moment. It is equivalent to an operator with one-step delay, which provides the whole network with the dynamic memory function. The mathematical model of Elman neuron network is as follows:

(13) $$x\left(k\right)=f\left[\omega_{ij}^{x}x^{c}\left(k\right)+\omega_{ij}^{u}u\left(k-1\right)\right]\;$$

(14) $$x^{c}\left(k\right)=\alpha x^{c}\left(k-1\right)+x\left(k-1\right)\;$$

(15) $$y\left(k\right)=g\left[\omega_{ij}^{y}x\left(k\right)\right],$$

where *u*(*k* − 1) is the input of the input layer node; *x*(*k*) is the output of the hidden layer node; *y*(*k*) is the output of the output layer node; *x_c_*(*k*) is the feedback state vector; $\omega_{ij}^{x}$, $\omega_{ij}^{y}$, and $\omega_{ij}^{u}$ are the connection weight matrices from the input layer to hidden layer, from associative layer to hidden layer, and from hidden layer to output layer, respectively; g(·) is the transfer function of neurons in the output layer; f(·) is the transfer function of neurons in the hidden layer, and Sigmoid function is usually used; α is the self-feedback gain factor, where 0 < *α* < 1.

In this study, the two-dimensional integrated feature information of motor operating states was predicted. The first 1234 points of feature parameters were used to train the Elman neuron network model, thus obtaining an Elman neuron network training model where 50 points were taken as the input and one point as the output. The data collected from *1235*-th to *1294*-th points served as the verification data to verify model precision and make parameter adjustment. The rest of the 60 points after the *1294*-th point were predicted using the aforementioned model. Root mean square error (RMSE) was used to predict the error between the predicted results and observed values based on the following formula [27]:

(16) $$RMSE=\sqrt{\frac{\sum_{i=1}^{n}{\left(X_{pre,i}-X_{obs,i}\right)}^{2}}{n}},$$

where *X_pre,i_* is the predicted value; *X_obs,i_* is the observed value; *n* is the number of points to be predicted. Prediction results are shown in Table 4.

Prediction results above suggested enhanced fusion feature prediction precisions of all four subspace learning algorithms after using UFS-MIFD for feature selection. This also indicated that UFS-MIFD contributed to the performance enhancement of subspace learning algorithms.

## 6. Conclusions

To overcome the information fusion performance decline of subspace learning algorithms caused by the redundant and irrelevant features in the multidimensional feature parameter set, the mutual information and fractal dimension-based unsupervised feature selection algorithm is studied. A UFS-MIFD method is proposed using various theories and methods, including original feature extraction method, mutual information, and fractal theory, in response to the long computing time, high time complexity, and the possibility of failing to identify the optimal solutions that plague previous unsupervised feature selection algorithms. With this method, a feature importance ordering algorithm that takes the relevance and redundancy of features into overall consideration is developed. The optimal feature subset is identified by eliminating the highly-redundant feature parameters with low relevance to the whole feature parameter set based on the fractal dimension-based feature subset evaluation criterion. In addition, a performance degradation test of brushless DC motor of multi-rotor UAV is designed to verify the proposed method based on the vibration signal data. To verify the proposed UFS-MIFD, the information fusion performance of subspace learning algorithms before and after the use of UFS-MIFD is compared by measuring the motor health-fault diagnosis capacity and motor state prediction effect. Comparison results suggest that UFS-MIFD can play a role in enhancing the information fusion performance of subspace learning methods. Not only is the proposed method able to reduce the negative influence of irrelevant and redundant features and excessive dimension on subsequent algorithms and decisions and enhance the precision and stability of subsequent research results, but it is also of high engineering value since it can be used for the feature selection of large volumes of unlabeled data. With limited data of the motor under test, however, there is still room for the improvement and optimization of the proposed method with the increase of test subjects and sample size. Moreover, because the application of the proposed method in this paper is specific, the proposed method can be applied to the feature selection of vibration signals of similar UAVs’ operating systems. In other words, it is not clear if the behavior of the proposed method will be the same for different types of signals of other applications. Therefore, the adaptability and universality of the proposed method will be further discussed and investigated in the following research.

## Figures and Tables

**Table 1:** Conditions of the motor performance degradation test.

Motor Model	KV Value	Voltage	Current	Throttle	Rotation Speed	Sampling Direction	Sampling Frequency	Blade
U8	170	22.2 V	27 A	100%	2300 rpm	*X*, *Y*, *Z* axes	12.8 kHz	28 × 9.2

KV value represents the increased speed per volt.

**Table 2:** Feature parameter subsets along *X*, *Y*, and *Z*-axes.

***X***	**1**	**2**	**3**	**4**	**5**	**6**	**7**
	Peak	Skewness	Pluse	Mean Value	HESE	Margin	WPBE5
	**8**	**9**	**10**	**11**	**12**	**13**	**14**
	RMS	ASE	WPBE1	WPBE4	WPBE2	FV	GF
	**15**	**16**	**17**				
	WPBE8	HMSE	WPBE6				
***Y***	**1**	**2**	**3**	**4**	**5**	**6**	**7**
	Peak	ASE	MSF	HMSE	RMS	WPBE4	WPBE6
	**8**	**9**	**10**	**11**	**12**	**13**	**14**
	WPBE5	Skewness	Margin	WPBE2	WPBE8	Peak Value	VAR
	**15**	**16**					
	Mean Value	HESE					
***Z***	**1**	**2**	**3**	**4**	**5**	**6**	**7**
	MSF	WPBE2	Skewness	VAR	Margin	WPBE4	ASE
	**8**	**9**	**10**	**11**	**12**	**13**	****
	WPBE3	Peak Value	RMS	Mean Value	WPBE7	FV	

**Table 3:** Evaluation of “health-fault” state diagnosis results based on integrated feature parameters before and after the use of UFS-MIFD.

Subspace Learning Method	KPCA	PCA	LPP	LDA
**Before the use of UFS-MIFD**	0.5488	2.4611	0.8833	2.7966
**After the use of UFS-MIFD**	0.5373	2.2265	0.2278	2.4750
**Percentage**	2.1%	9.53%	74.21%	11.50%

**Table 4:** Comparison between the predicted and observed values of the two-dimensional integrated feature parameter states before and after the use of UFS-MIFD.

Subspace Learning Method	KPCA		PCA		LPP		LDA	
	1st Feature	2nd Feature	1st Feature	2nd Feature	1st Feature	2nd Feature	1st Feature	2nd Feature
**Before the Use of UFS-MIFD**	0.3291	0.3077	0.7940	0.4351	1.1280	0.4888	16.3521	8.7041
**After the Use of UFS-MIFD**	0.3175	0.2740	0.6370	0.3205	1.0609	0.4420	12.5659	6.4507
